# The progression in atrial fibrillation patients with COPD: a systematic review and meta-analysis

**DOI:** 10.18632/oncotarget.22092

**Published:** 2017-10-26

**Authors:** Xiaoying Chen, Meiling Lin, Wei Wang

**Affiliations:** ^1^ Department of Cardiology, The Second Affiliate Hospital of Shantou University Medical College, Guangdong, 515000, China; ^2^ Department of Cardiology, The First Affiliate Hospital of Shantou University Medical College, Guangdong, 515000, China

**Keywords:** COPD, atrial fibrillation, progression, recurrence, meta-analysis

## Abstract

**Aim:**

Chronic Obstructive Pulmonary Disease (COPD) and atrial fibrillation (AF) share pathophysiological links, as supported by the high prevalence of AF within COPD patients. AF progression and recurrence can increase the risks of mortality, morbidity and adverse cardiovascular events. The present systematic review and meta-analysis aims to assess the risk for AF progression and recurrence for COPD patients, to further demonstrate the risk of COPD in AF patients.

**Methods and Results:**

A systematic review was conducted in MEDLINE / PubMed and Cochrane Library and Embase, Web of science. Prospective studies including AF patients with COPD were screened and included if matching inclusion and exclusion criteria. 7 studies were included, adding up to 10761 AF patients (1556 with AF and COPD, 9205 without COPD). Mean age from each study ranged from 51 to 81 years, and 57.2% were male. Hypertension accounted for 75.5% of the population, and 20.7% had the comorbidity of diabetes mellitus. The pool analysis showed that COPD could promote AF progression (OR = 1.90; 95% CI, 1.34–2.68, I^2^ = 77%, *p* = 0.0003). For subgroup analysis, we found that COPD could increase the risk of AF recurrence (OR = 2.35; 95% CI, 1.86–2.97, I^2^ = 0%, *p* = 0.39). Besides, in the younger group, at the median age of 64, COPD was still a risk factor for AF progression (OR = 2.22; 95% CI, 1.80–2.74, I^2^ = 0%, *p* = 0.69).

**Conclusions:**

COPD is an independent risk for AF progression and recurrence, COPD patients with AF carry a worse prognosis than those in sinus rhythm (SR).

## INTRODUCTION

Atrial fibrillation (AF) is the most common form of chronic arrhythmia, with increasing health care burden and the mainly complications of atrial fibrillation are stroke and heart failure. Various studies had demonstrated that AF progression from the paroxysmal atrial fibrillation (PAF) to more sustained forms, and recurrence could increase morbidity and mortality. AF patients with progression have more adverse cardiovascular events and are more often admitted in the hospital [1]. The higher HATCH (hypertension, age > 75 years, previous transient ischemic attack or stroke chronic obstructive pulmonary disease, heart failure) scores correlate with higher occurrence of AF and a high risk for stroke, which can be used as a selection criteria for intensified Electrocardiograph monitoring [2].

Chronic obstructive pulmonary disease (COPD) is a major public health problem and is characterized by non-reversible airflow limitation. COPD is not only an independent predictor for major adverse cardiac events but also a predictor of AF incidence [3, 4]. The rate of AF incident was inversely associated with forced expiratory volume in one second (FEV1). AF prevalence was higher in severe airflow obstruction subject than those with mild or moderate airflow obstruction [5, 6]. Moreover, the risk of AF hospitalization was higher among the lower FEV1, especially for FEV1 < 60% [7]. Previous Studies have reported that the major cause of deaths in COPD patients is cardiovascular diseases (CVD) rather than respiratory failure and it is more likely to develop acute coronary syndrome and heart failure. Furthermore, COPD contributes most to the increased all-cause mortality of AF [8].

Various mechanisms about the association between AF and COPD had been widely explored. Oxidative stress and chronic systemic inflammation play vital roles in the pathology of COPD, resulting in atrial myocyte breakdown and fibrosis, and as a consequence progress to AF initiation and maintenance. Glycogen accumulation and atrial fibrosis are proved be risk factors for AF progression in animal models [9]. In the hypoxia condition among the COPD patients, matrix metalloproteinases expression increase which is a important molecular for the regulation of atrial structural remodeling [10]. Hypoxia can cause the constriction of pulmonary arteriolar gradually resulting in the pulmonary hypertension. Higher PaCO2 values and higher pulmonary artery systolic pressure (PASP) value significantly raise the AF prevalence [11]. Furthermore, low potassium is one of most common electrolyte disturbances in COPD, which might be caused by the use of corticosteroids or beta-blockers. The serum potassium level can influence the cell membrane potential. Low serum potassium increases the p-wave duration which is also a predictor for the incidence of AF [12].

Pulmonary hypertension can contribute to right atrial (RA) hemodynamic overloading or stretching, resulting in the prevalence of non-pulmonary vein (PV) foci in the COPD patients [9]. Commonly, most PAF originate from the pulmonary veins (PVs).There are still some other originations in the non-PV foci such as the superior vena cava (SVC), left atrial free wall (LAFW), crista terminalis (CT), coronary sinus ostium, ligament of Marshall, left atrial appendage, and interatrial septum which have been proved to be a risk factor for AF progression and recurrence. Among these patients the non-PV triggers could not be incompletely eliminated. Moreover, the RA conduction time is prolonged with COPD and the prevalence of typical atrial flutter recurrence increases positively with the severity of COPD, regardless of effective therapies [13].

Although many traditional factors that cause heart structural remodeling such as hypertension, age, heart failure, diabetes can promote AF progression and recurrence, there is few data concerning the influence of COPD on progression and recurrence in all kinds of AF patients [14].

A wide range of methods including catheter ablation, pharmacological cardioversion, electrical cardioversion are used for the maintenance of sinus rhythm in AF patients. The AF recurrence and progression rates are still high causing a heavy economic burden. Therefore, the present systematic review and meta-analysis aims to investigate the role of predictor that COPD plays for AF.

## MATERIALS AND METHODS

### Search strategy and criteria

Firstly, we searched MEDLINE/PubMed, EMBASE and Cochrane databases and Web of Science. The following MeSH, full text and keyword terms: “atrial fibrillation” AND (“COPD” OR “chronic obstructive pulmonary disease” OR “Pulmonary Disease, Chronic Obstructive”) were used to identify all the published articles in English. We excluded the trials published solely in abstract form because the results could not be fully analyzed.

### Data extraction

Two blinded reviewers (Xiaoying Chen and Meiling Lin) first screened independently the citations. The specific inclusion criteria were: (1) human studies, (2) the investigated patients are AF patients which was not developed after the CABG, (3) included the outcome of atrial progression and recurrence, (4) the mean follow-up of at least 6 months, (5) progression was defined as change from paroxysmal or persistent AF to a more sustained form, (6) recurrences were defined as episodes of AF or atypical atrial flutter or atrial tachycardia lasting at least 30s detected during follow-up at least after 6 months. Exclusion criteria: (1) non-human studies, (2) studies including patients undergoing CABG atrial fibrillation, (3) valvar atrial fibrillation.

### Statistical analysis

Since none of the included studies was random control trail, we assessed risk of bias using the Newcastle-Ottawa scale. The pooled Odds ratio and 95% confidence intervals were calculated using the Mantel-Haenszel method. A fixed-effect model was used if the trials were similar, and a random-effects model was used if they varied. The numbers with progression or recurrence were calculated. Plot analysis was used to evaluate potential publication bias, and Cochran *Q*^2^ tests and I^2^ to investigate heterogeneity.

## RESULTS

### Search results

**Figure 1:**
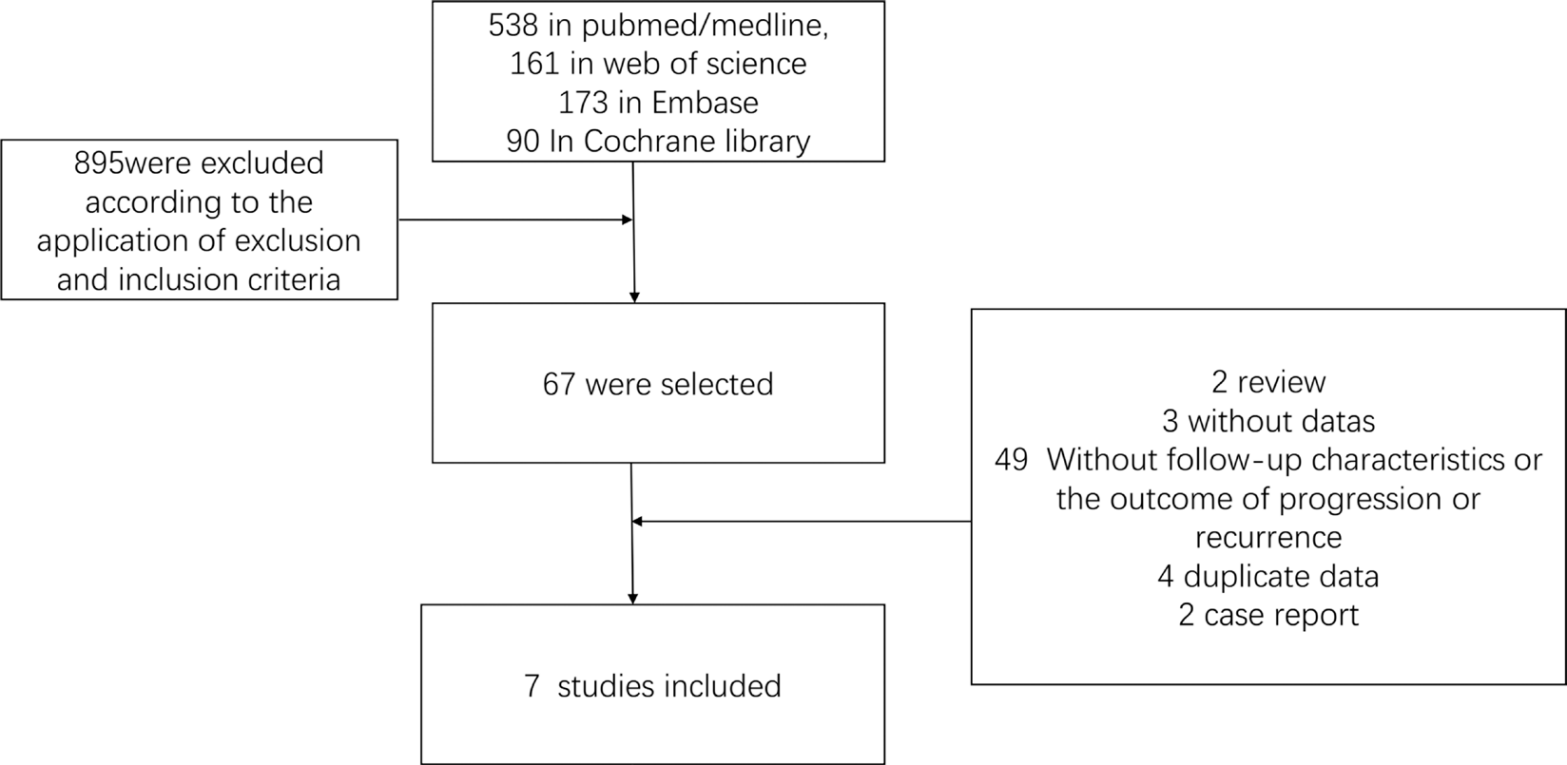
Search criteria and flow graph of the studies screened and included in this meta-analysis

The searching strategies in different database identified 962 abstracts (538 in PubMed/MEDLINE, 161 in Web of Science 173 in EMBASE, 90 In Cochrane library. Among them 895 were excluded according to the application of exclusion and inclusion criteria. 67 of them were selected and full text was read by two blinded authors. Among them, 2 were case reports and 3 were without data. 49 were excluded because of incomplete baseline, follow-up characteristics, or without the outcome of progression or recurrence. 4 were excluded because of reporting duplicate data and 2 because it was a case report. Following this selection approach, 7 trials were identified and finally 7 studies were included in the meta-analysis (Figure 1).

### Baseline patient characteristics

Our literature search identified 7 studies investigating the potential association between COPD and AF recurrence and progression despite of different cardioversion. A total of 10761 patients with AF were finally included from 7 studies.(1556 with AF and COPD, 9205 without COPD). Based on the 6 studies reporting this detail, 63.9% were paroxysmal AF, 22.8% persistent AF patients, 6.7% long-standing persistent AF and 5% first detected AF. Baseline characteristics are shown in Table 1 and Table 2. Mean age from each study ranged from 51 to 81 years, and 57.2% were male. Hypertension accounted for 75.5% of the population. And 20.7% had the comorbidity of diabetes mellitus (DM). The mean left atrial diameter was 43.3 mm, and mean BMI was 27.4 kg/m^2^. 4 studies provided specific therapy [13, 15–17]. Among them, 30.2% were treated with electrical cardioversion, 45.7% with antiarrhythmic drugs, and 24.4% with catheter ablation. As all the studies which were included in this analysis were prospective studies, the Newcastle Ottawa Scale (NOS) was used to assess the methodological quality of the studies. NOS assessment involved a minimum number of zero to a maximum number of 8 depending on the quality of the study being assessed. The results have been listed in Table 3.

**Table 1:** First author, publication date, population, and main characteristics of the included studies

Author	year	study design	Study population	Follow-up (months)	Number of patients	Mean age (years)	Male	Hypertension	DM
Pisters, Ron [15]	2012	prospective study	1801	12	1801	64.1	1063	1149	304
Okcun, B. [16]	2002	prospective study	173	6	110	69 ± 9	56	70	15
Hayashi, Takekuni [13]	2013	prospective study	181	> 6	68	64.5	54	N/A	N/A
Gu, J. [17]	2013	prospective study	550	31.4 ± 4.8	550	64.2	326	290	55
de Vos, C. B. [19]	2010	prospective study	1219	12	1219	64 ± 13	695	752	182
Holmqvist, F. [29]	2015	prospective study	6235	6	6235	74 (65–81)	3554	5113	1746
Vidal-Perez, R. [24]	2013	prospective study	788	33.6 ± 8.4	788	74.8 ± 9.2	413	595	192

DM. Diabetes Mellitus; N/A, no available.

**Table 2:** Baseline characteristics of study population included

Author	year	Mean AF duration (months)	Paroxysmal	persistent	long-standing	First detected	BMI (kg/m^2^)	Left atrial diameter (mm)	ECV	Drug	Catheter ablation
Pisters, Ron	2012	N/A	631	653	no	493	27.8	44.2	712	1098	no
Okcun, B.	2002	89.1 ± 75.3	N/A	N/A	N/A	N/A	N/A	N/A	52	58	no
Hayashi, Takekuni	2013	44.4 ± 56.3	36	24	8	no	N/A	40.0 ± 7.4	no	no	68
Gu, J.	2013	59.1 ± 13.1	191	189	170	no	24 ± 2.5	46.2 ± 3.2	no	no	550
de Vos, C. B.	2010	N/A	1054	no	no	165	27 ± 4	43 ± 8	N/A	N/A	N/A
Holmqvist, F.	2015	42 (18–85)	4739	1496	no	no	29 (25–34)	43 (38–48)	N/A	N/A	N/A
Vidal-Perez, R.	2013	73.2 ± 61.2	106	55	529	88	30.1 ± 4.8	N/A	N/A	N/A	N/A

N/A, no available; AF, atrial fibrillation; BMI, body mass index; ECV electrical cardioversion.

**Table 3:** The Newcastle-Ottawa Scale (NOS) for assessing the quality of the studies

Study	Selection				Comparability	Outcome			Total
	Representativeness of the exposed cohort	Selection of the non exposed cohort	Ascertainment of exposure	Demonstration that outcome of interest	basis of the design or analysis	Assessment of outcome	follow-up long enough for outcomes	Adequate follow up	
Pisters, Ron [15]	1	1	1	1	1	1	1	1	8
Okcun, B [16]	1	1	1	1	1	1	1	1	8
Hayashi, Takekuni [13]	1	1	1	1	1	1	1	1	8
Gu, J [17]	1	1	1	1	1	1	1	1	8
de Vos, C. B [19]	1	1	1	1	1	1	1	1	8
Holmqvist, F [29]	1	1	1	1	1	1	1	1	8
Vidal-Perez, R [24]	1	1	1	1	1	1	1	1	8

### AF recurrence and progression associated with COPD

**Figure 2:**
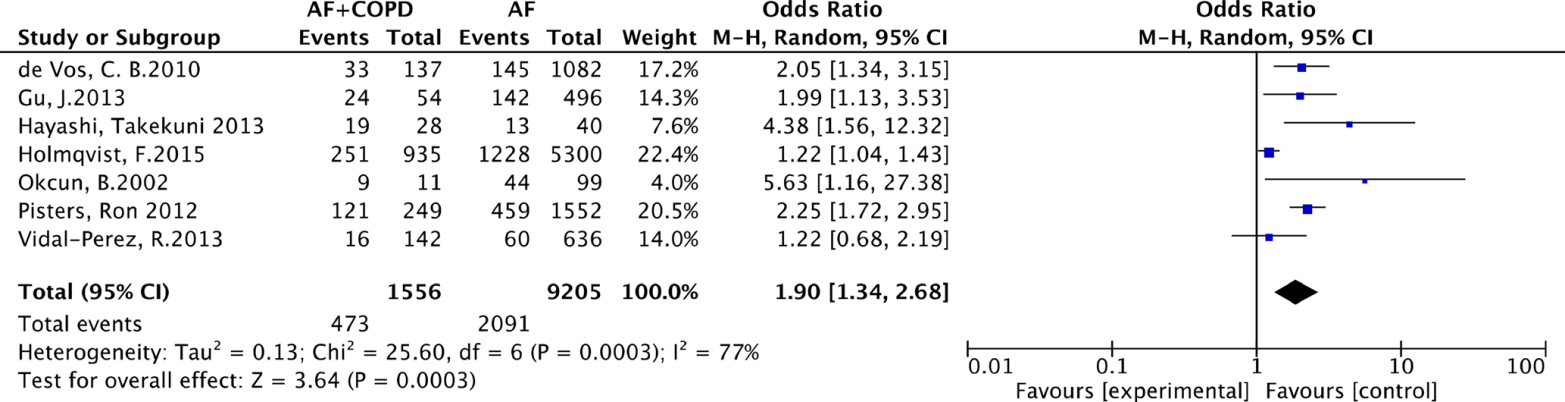
Forest plot on the association between COPD and atrial fibrillation recurrence and progression

**Figure 3:**
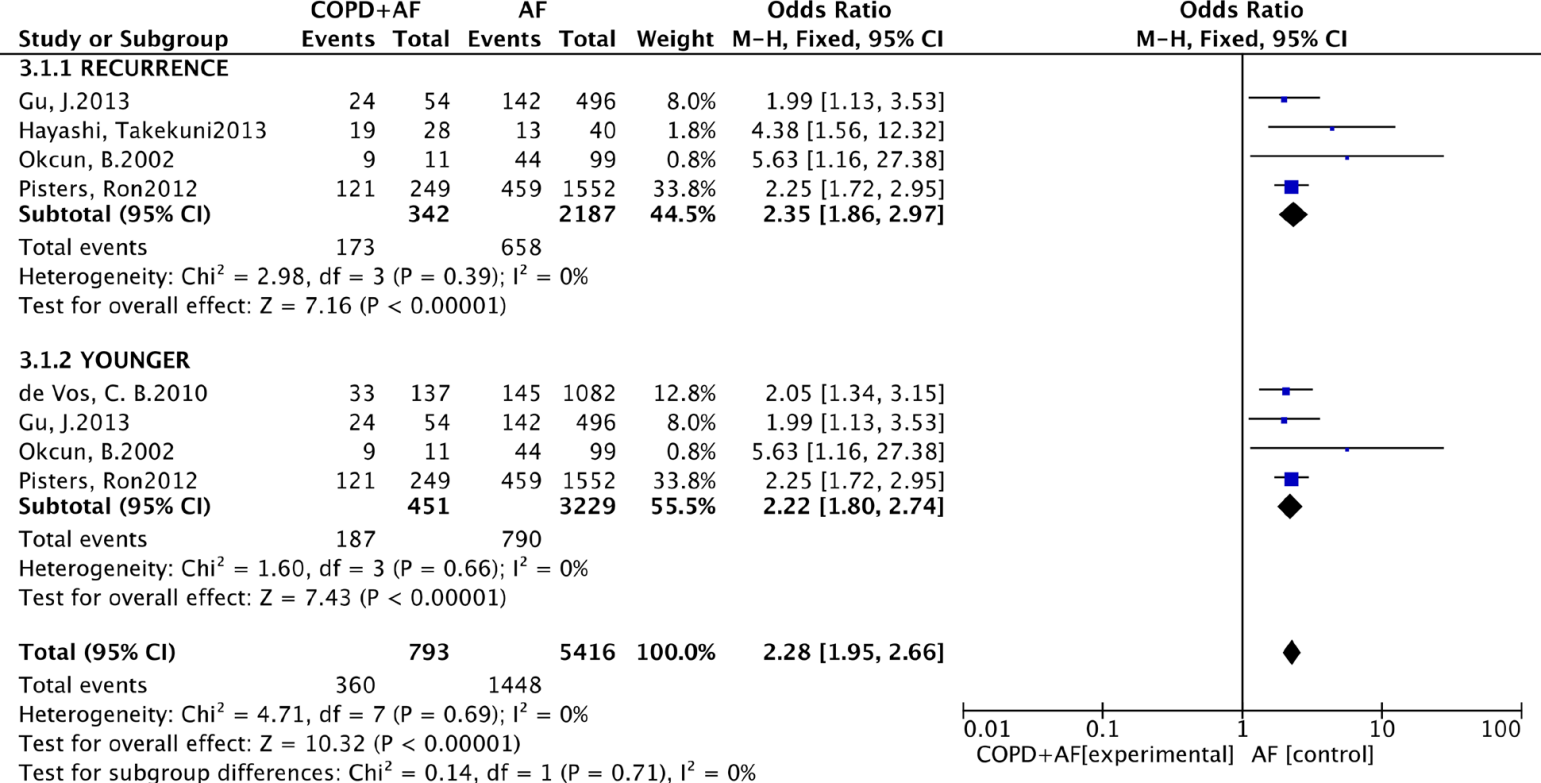
Subgroup analysis: forest plot on the association between COPD and atrial fibrillation recurrence

**Figure 4:**
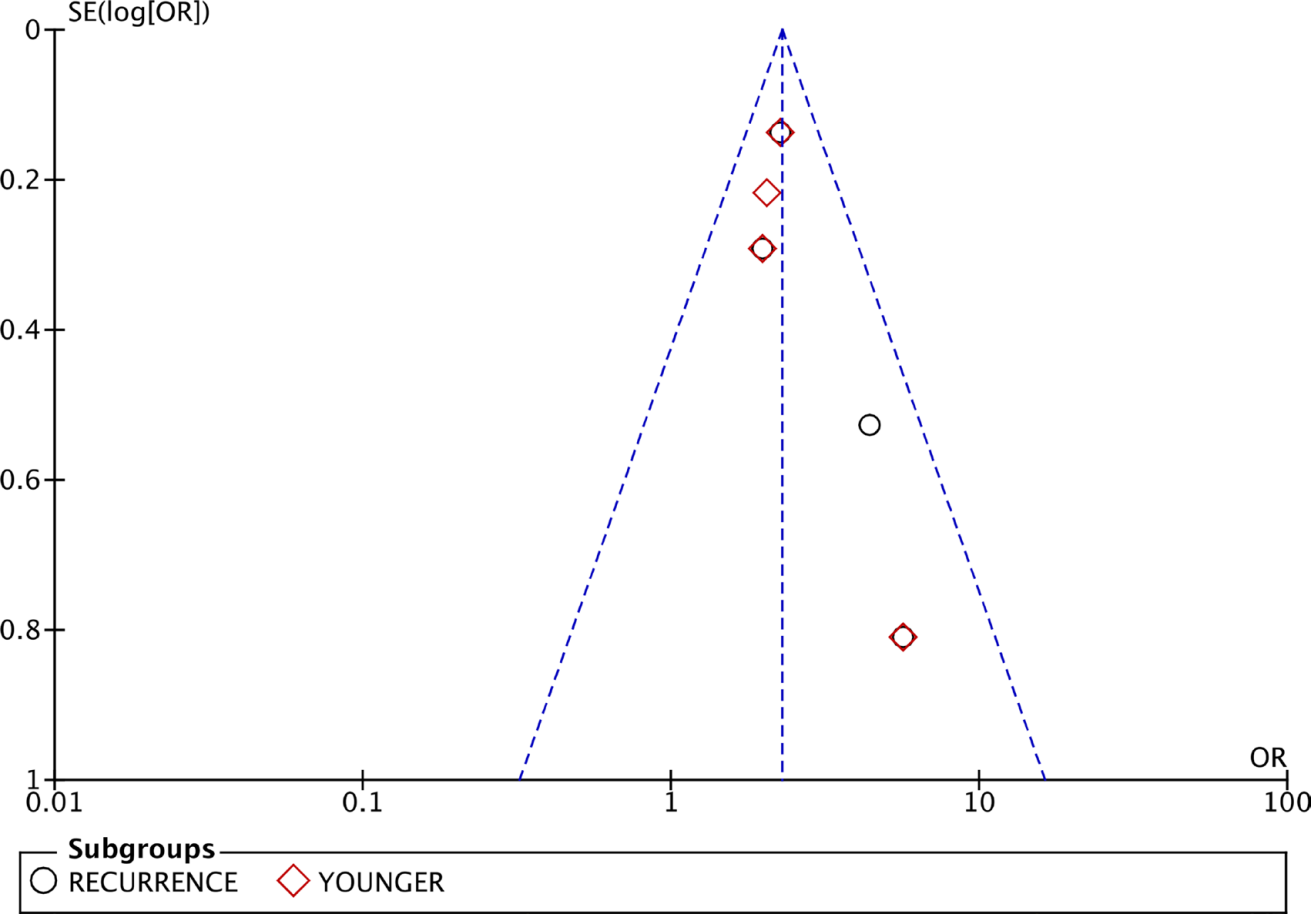
Funnel plot to analyze publication bias

As listed in Figure 2, the pool analysis showed that COPD could promote AF progression (OR = 1.90; 95% CI, 1.34–2.68, I^2^ = 77%, *p* = 0.0003). For subgroup analysis (Figure 3), we also found COPD could increase the risk of AF recurrence (OR = 2.35; 95% CI, 1.86–2.97, I^2^ = 0%, *p* = 0.39). Among all the included studies, in the younger ages, at the median age of 64, COPD was still a risk factor for AF progression (OR = 2.22; 95% CI, 1.80–2.74, I^2^ = 0%, *p* = 0.69). Analysis of publication bias was conducted. The funnel plots showed a low to moderate risk of publication bias across the studies based on a visual analysis (Figure 4).

## DISCUSSION

COPD had been demonstrated increasing the risk of cardiac arrhythmia, especially for AF. AF patients with COPD often present more comorbid diseases, and at a higher risk of complications [18]. Among all the present studies concluded, the majority of classified deaths of AF are cardiovascular diseases, whereas non-hemorrhagic stroke or systemic embolism constitute a small part. A 12-year follow-up study had proved that AF progression could increase hospital admissions and major adverse cardiovascular events [19].

Besides, anticoagulation therapy can improve survival in these patients, whether new oral anticoagulation or vitamins k antagonist [20]. Despite the high prescription of oral anticoagulation (OAC), 1-year mortality and morbidity remain high in AF patients, particularly from heart failure and hospitalizations [21]. Moreover, anticoagulation treatment is inadequate in patients with AF and COPD [22]. It had been proved that COPD, cancer and chronic heart failure (CHF) were each associated with over 20% increased risk of OAC treatment discontinuation [20, 23].

COPD patients showed a higher trend to concomitant with structural heart disease. Our data conformed the positive association between AF progression and high mortality and morbidity [24]. COPD is also a vital risk factor for AF patients in terms major adverse cardiac events (MACE) including all-cause mortality, stroke, non-central nervous system systemic embolism and major bleed [25]. The cardiovascular and non-cardiovascular death were higher among AF patients with COPD [16]. However, there are no unanimous results about whether COPD could increase the risk of stroke or systemic embolism in AF patients. Some studies figured out that the presence of COPD might increase the occurrence of ischemic stroke in subjects with AF, while some other studies did not find [26, 27].

Actually, long-term SR maintenance is an effective treatment to reduce the risk of complications related to AF and the reasonable control of ventricular rate can increase the survival rate [28]. Many patients with PAF included in this study progressed to persistent or permanent AF, despite hard effect to maintain sinus rhythm [19]. The absence of COPD is a predictor of the maintenance of sinus rhythm after electrical cardioversion or pharmacological cardioversion whether it is oral or intravenous chemical cardioversion [15]. Higher heart rates and advanced ages are also strong predictors for AF progression from paroxysmal or persistent a more sustained form [29, 30].

Catheter ablation of AF is widely applied for patients with symptomatic AF. COPD can affect lung morphological alterations which will cause marked change in PV anatomy, increasing the risk of AF recurrence. Non-pulmonary vein foci in the right atrial are also common in AF patients with COPD. The present study confirmed that catheter ablation of AF is feasible and safe in COPD patients using Bispectral index (BIS) monitoring to ablation [31].

Catheter ablation of AF can reduce AF progression risk [32]. Sometimes, prophylactic cavotricuspid isthmus ablation is needed to combine with PV isolation, which may be considered for patients with COPD. However, the recurrence of arrhythmia after first ablation is higher in this type of patients and most of them needed to be performed a second ablation [13, 33, 34].

Besides, AF will influence the drug treatment of COPD. Beta-adrenergic agonist and theophylline are usually used in the acute exacerbation of COPD, which can accelerate the ventricular rate in AF patients and might deteriorate the circulation stability. Salbutamol can increased heart rate, a strong predictor of AF progression [35]. The 2016 ESC guideline had clearly figured out that non-selective beta-blockers, propafenone and adenosine should be used with caution in patients with significant bronchospasm. Among AF patients with COPD beta-1 selective blockers like bisoprolol, metoprolol, are safe [36]. Amiodarone was the most widely used among all the antiarrhythmic drugs in the persist AF [37]. Antiarrhythmic drugs used as rhythm or rate control agents in the treatment of AF have been associated with a reduction in cardiovascular but not in all-cause mortality, which may seem reasonable considering their mode of action.

As for the predictor of the successful electrical cardioversion, the absence of COPD, chronic heart failure, obstructive sleep apnea syndrome (OSAS), valvular disease, higher HATCH scores, larger left atrial diameter, lower ejection fraction(EF) and advanced ages remained significant [17].

## CONCLUSIONS

AF progression and recurrence in patients with COPD are of higher risks for mortality, stroke and MACE. COPD is an independent risk for AF progression and recurrence. COPD patients with AF carry a worse prognosis than those in SR. Therefore, to maintain SR long-term may help to reduce the negative impact of AF in COPD patients with effective rhythm control treatments.

### Limitations

This study presents the following limitations. First, the present studies include all the treatment of AF including the catheter ablation, electrical cardioversion and drug cardioversion to evaluate the outcome. However, the choose of therapy is based on the overall evaluation of the patients’ physical condition and most of the medication-refractory AF will undergo catheter ablation, which might cause selection bias. Additionally, most patients are not only combined with COPD. Patients with AF and COPD have more traditional cardiovascular risk factors (diabetes mellitus, hypertension, aging, heart failure, impaired renal function, obesity, larger LA diameter), all of which are important parameters that predict recurrence and progression. These complicated clinical features may weaken the predictive effects of comorbidity COPD on progression and recurrence. Thirdly, in order to include the largest dates, almost all of them are observed studies and prospective studies instead of random control trials. There might be some selection bias.
